# Research on multi-object detection technology for road scenes based on SDG-YOLOv5

**DOI:** 10.7717/peerj-cs.2021

**Published:** 2024-04-30

**Authors:** Zhenyang Lv, Rugang Wang, Yuanyuan Wang, Feng Zhou, Naihong Guo

**Affiliations:** 1 School of Information Technology, Yancheng Institute of Technology, Yancheng, China; 2 Yancheng XiongYing Precision Machinery Company Limited, Yancheng, China

**Keywords:** Intelligent driving, Road scene detection, Decoupled detection head, Attention mechanism

## Abstract

To resolve the challenges of low detection accuracy and inadequate real-time performance in road scene detection, this article introduces the enhanced algorithm SDG-YOLOv5. The algorithm incorporates the SIoU Loss function to accurately predict the angle loss of bounding boxes, ensuring their directionality during regression and improving both regression accuracy and convergence speed. A novel lightweight decoupled heads (DHs) approach is employed to separate the classification and regression tasks, thereby avoiding conflicts between their focus areas. Moreover, the Global Attention Mechanism Group Convolution (GAMGC), a lightweight strategy, is utilized to enhance the network’s capability to process additional contextual information, thereby improving the detection of small targets. Extensive experimental analysis on datasets from Udacity Self Driving Car, BDD100K, and KITTI demonstrates that the proposed algorithm achieves improvements in mAP@.5 of 2.2%, 3.4%, and 1.0% over the original YOLOv5, with a detection speed of 30.3 FPS. These results illustrate that the SDG-YOLOv5 algorithm effectively addresses both detection accuracy and real-time performance in road scene detection.

## Introduction

With the substantial rise in global vehicle ownership in recent years, there an increasing pressure on road traffic resulting in a higher incidence of accidents. This highlights the necessity for the development of effective intelligent driving systems to reduce the risk of traffic incidents. Autonomous vehicles, which integrate various intelligent technologies, primarily rely on a range of sensors and systems including ultrasonic radars, millimeter-wave radars, LiDAR, and camera vision sensors to accurately perceive and interpret the surrounding environment and obstacles (Xu et al., 2022b; Xu et al., 2022a; Du et al., 2022; Lin et al., 2022). Among these sensors, low-cost camera vision sensors with integration mechanisms have become a research hot spot in research focusing on detecting, recognizing, and tracking road targets. In this context, target detection technology has emerged as a central area of study in the intelligent driving domain, playing a fundamental role in camera vision sensors. It predominantly employs deep learning algorithms to analyze image or video data, effectively identifying and locating various target objects such as pedestrians, vehicles, and traffic signs within the visual content. This process is critical for achieving reliable environmental perception in intelligent driving systems as it directly influences decision-making and control mechanisms, ensuring safe and efficient vehicle navigation. Presently, target detection algorithms are primarily classified into traditional methods and those utilizing deep learning techniques (Liu et al., 2023b; Luo et al., 2022; Zhang et al., 2023; Zhou et al., 2018; Zhou et al., 2021a). With the rapid advancements in computer technology, deep learning-based target detection methods have attracted scholars due to their high accuracy and real-time processing capabilities. However, the presence of numerous challenging-to-detect targets in complex traffic environments adversely affects the detection accuracy of conventional algorithms. Additionally, many high-precision algorithms exhibit practical limitations in real-time tasks, failing to meet the demands of intelligent driving. Accordingly, exploring ways to improve the precision of target detection while maintaining real-time processing is of significant importance in the intelligent driving field.

## Related Work

Deep learning object detection algorithms are primarily classified into one-stage and two-stage categories based on different detection principles. One-stage object detection algorithms, exemplified by YOLO (Redmon et al., 2016) and SSD (Liu et al., 2016), employ regression strategies for object detection. Two-stage object detection algorithms are represented by R-CNN (Girshick et al., 2014), SSP-Net (He et al., 2015), and Fast R-CNN (Liu et al., 2022). On the other hand, two-stage object detection algorithms, represented by R-CNN (Girshick et al., 2014), SSP-Net (He et al., 2015), and Fast R-CNN (Liu et al., 2022), generate proposal regions based on the original image, utilize convolutional neural networks for feature extraction, and subsequently perform object classification and detection. Although two-stage object detection algorithms, which scan all image areas and process candidate boxes afterward, exhibit superior performance, these algorithms lack real-time capability, thereby limiting their application in intelligent driving systems. On the contrary, one-stage detection algorithms are end-to-end approaches based on bounding box regression. These algorithms do not necessitate generating numerous candidate regions, thus significantly reducing detection time and enhancing the real-time performance of object detection. These algorithms offer certain advantages in engineering applications and have shown considerable research progress. For instance, Redmon et al. (2016) introduced the YOLOv1 algorithm, which utilizes global information from an image for predictions. Nevertheless, its limitation in predicting only two boxes of the same class per grid reduces its effectiveness in detecting closely situated objects and small targets. Subsequently, Redmon & Farhadi (2017) modified the YOLOv1 algorithm and developed YOLOv2 and YOLO9000 algorithms, introducing an anchor mechanism that significantly improved the recall rate. However, detecting small objects remained a challenge. To resolve this challenge, YOLOv3 was developed, incorporating a feature pyramid network (FPN) to fuse features across multiple scales (Redmon & Farhadi, 2018). This algorithm, utilizing DarkNet-53 as its backbone network and multiscale prediction in the output section, enhanced the accuracy of detecting smaller targets. Additionally, it replaced softmax with logistic regression to improve accuracy while maintaining real-time capabilities, although its accuracy was still insufficient for industrial applications. Further advancements were made with the introduction of YOLOv4 (Bochkovskiy, Wang & Liao, 2020), which utilized the FPN+PAN structure to enhance feature communication and employed CSPDarkNet-53 as the backbone network, resulting in improved detection speed and accuracy. Cai et al. (2021) proposed YOLOv4-5D, focusing on intelligent driving and enhancing small target detection accuracy by utilizing CSPDarknet53-dcn as the backbone network and introducing the PAN++ feature fusion module along with expanding to five detection layers. Moreover, Li et al. (2022) introduced YOLOv6, designed for industrial applications, integrating the RepVGG re-parameterization structure to expedite inference without compromising performance. Similarly, Wang, Bochkovskiy & Liao (2023) proposed YOLOv7, incorporating techniques like E-ELAN, RepVGG structure, and SimOTA. Recently, Wang et al. (2023) introduced CenterNet-Auto, which integrated the RepVGG architecture into the backbone network, combined feature pyramids, and employed deformable convolutions post-backbone. They also introduced an average boundary model to address occlusion challenges in intelligent driving. Despite significant advancements in enhancing detection accuracy through recent algorithmic developments, two notable disadvantages have emerged: increased training resource consumption and reduced detection speed. Incorporating structures like RepVGG and SimOTA has prolonged training time due to increased network complexity, while the use of deformable convolutions has escalated computational demands without achieving an optimized balance between real-time performance and detection accuracy. Therefore, achieving this balance is crucial in the field of intelligent driving.

Based on the conducted literature review, this study employs YOLOv5 as the base model for improvement, ignoring newer algorithms like YOLOv6, v7, and v8. This decision was made considering various factors such as overall performance, efficiency, maturity, and community support. While YOLOv6 exhibits notable detection capabilities with the RepVGG structure, it introduces hardware dependencies and resource-intensive training. Furthermore, YOLOv7’s enhancements in precision through E-ELEN and MP structures come at the expense of increased model parameters, impacting efficiency. In contrast, YOLOv5 offers a balance of maturity and community backing, making it the preferred choice for developing a stable and widely supported algorithm. Accordingly, the article introduces SDG-YOLOv5, an algorithm developed for road scene detection. Addressing challenges such as target diversity and background complexity, the article proposes the SIoU Loss function to optimize angular loss in prediction boxes, thereby enhancing regression precision and convergence speed. Additionally, to resolve focus conflicts inherent in traditional coupled detection heads, lightweight decoupled heads (DHs) are devised to separate classification and regression tasks and improve detection accuracy and efficiency. Furthermore, considering the intricacies of road scenes and the need for algorithms to grasp contextual nuances and object relationships, the article introduces a lightweight Global Attention Mechanism Group Convolution (GAMGC). This mechanism allows the algorithm to capture more contextual information, enhancing object recognition, especially in complex scenarios like occlusions or varying lighting conditions. Finally, experiments were conducted on various datasets, including Udacity Self Driving Car, BDD100K, and KITTI to evaluate the effectiveness of the developed algorithm in road scene detection.

## Methodologies

Considering the variations in model structure depth and width, the YOLOv5 series is categorized into four versions based on the scale: YOLOv5-s, YOLOv5-m, YOLOv5-l, and YOLOv5-x. Among these versions, YOLOv5-s stands out with the smallest parameter count, making it ideal for applications requiring rapid response times. Accordingly, this study selects YOLOv5-s as the basic model to meet strict real-time performance demands. As the compact and efficient iteration within the YOLOv5 series, YOLOv5-s significantly enhances detection accuracy and robustness while satisfying real-time requirements through the integration of various innovative modules. Architecturally, the model adopts a Feature Pyramid Network (FPN) + Path Aggregation Network (PAN) for multi-scale feature fusion, optimizing the collaboration between the backbone and neck networks. Furthermore, cross-level feature extraction and multi-scale feature aggregation are accomplished through the inclusion of the CBS module, C3 module, and SPP module. The model adjusts the sizes of anchor boxes and feature map inputs, employing three coupled heads to respectively produce outputs for targets of varying scales, ensuring precise detection of both small and large objects. Moreover, at the input stage, the algorithm employs a range of data augmentation techniques like Mosaic and Mixup to dynamically adjust input images, thereby increasing the diversity of training data and bolstering the model’s generalization capability.

For road scene detection in intelligent driving applications, although the YOLOv5 algorithm demonstrates impressive detection speed, it should be optimized in terms of detection accuracy. The algorithm exhibits several deficiencies, as follows:

• The utilization of Complete-IoU Loss (CIoU Loss) in the network initially showed promise but was found to have a slower convergence rate. Moreover, it degrades to Distance-IoU Loss (DIoU Loss) as the aspect ratio of the predicted bounding box approaches that of the ground truth bounding box (Zheng et al., 2020). This degradation poses a concern as it could negatively impact the convergence speed and the accuracy of the model. Unlike CIoU Loss, DIoU Loss lacks penalties for discrepancies in aspect ratio and center points between predicted and ground truth boxes. Consequently, the model may converge prematurely or fail to adequately penalize localization errors during training, thereby compromising the final detection performance.
• The original network design incorporates a coupled detection head, merging the classification and regression tasks. While this design simplifies the network architecture, it introduces several drawbacks. Notably, coupling the tasks can lead to interference due to inherent differences in the objectives and optimization paths of classification and regression. The classification task aims to identify object categories, whereas regression focuses on accurately localizing object bounding boxes. When both tasks share the same detection head, the model may struggle to optimize conditions for each task simultaneously, compromising their respective accuracies. Additionally, this coupled design may restrict the model’s adaptability and tunability for individual tasks, making task-specific optimization more challenging and further impacting detection performance.
• Within the YOLOv5 network structure, the prominent utilization of CBS and C3 configurations is remarkable. These configurations employ convolutional operations to generate a substantial number of feature maps. While effective in extracting diverse feature information, this approach presents a notable issue: the creation of redundant feature maps. This redundancy increases the computational costs on the network and masks crucial information, potentially undermining the model’s accuracy in certain tasks.

**Figure 1:**
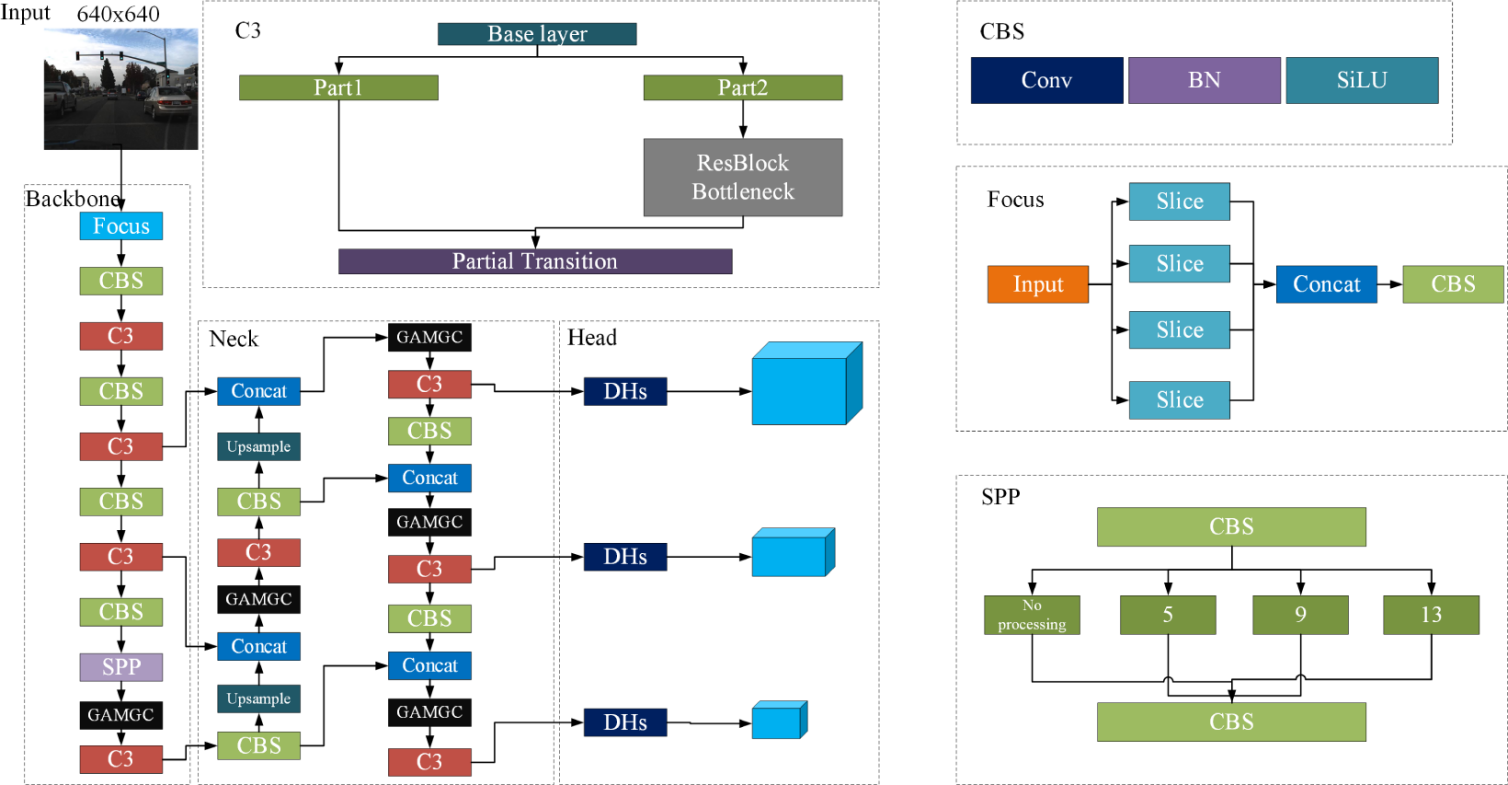
SDG-YOLOv5 network structure.

This section focuses on modifying the traditional YOLOv5-s structure. These improvements involve refining the loss function, optimizing the detection head, and incorporating an attention mechanism. The main objective is to address issues like low detection accuracy and inadequate real-time performance in road scene detection for intelligent driving. The updated network is named SDG-YOLOv5, and its structure is depicted in Fig. 1.

### Optimization of loss function

The overall loss function of the YOLOv5 detection algorithm combines Confidence Loss, Classification Loss, and Localization Loss, which are weighted and summed together. Specifically, for the bounding box regression loss function, CIoU Loss is utilized in the form below:

(1) \begin{eqnarray*}{L}_{CI\text{o}U}=1-I\text{o}U\text{+} \frac{{\rho }^{2} \left( b,{b}^{gt} \right) }{{c}^{2}} \text{+}\alpha \upsilon \end{eqnarray*}

where $I\text{o}U= \frac{ \left\vert B\cap {B}^{gt} \right\vert }{ \left\vert B\cup {B}^{gt} \right\vert } $ represents the intersection ratio between the predicted bounding box *B* and the actual bounding box *B*^*gt*^, $\rho \left( b,{b}^{gt} \right) =d$ is the Euclidean distance separating the center point of *B* and *B*^*gt*^, *c* denotes the diagonal distance of the smallest bounding box that wraps around *B* and *B*^*gt*^, *α* is the weight parameter, which is defined as follows:

(2) \begin{eqnarray*}\alpha = \frac{\upsilon }{ \left( 1-I\text{o}U \right) +\upsilon } ~\end{eqnarray*}

where *υ* is the aspect ratio, which is employed to evaluate the conformity of the aspect ratio between *B* and *B*^*gt*^. When the center points of *B* and *B*^*gt*^ coincide, *υ* can be expressed in the form below:

(3) \begin{eqnarray*}\upsilon = \frac{4}{{\pi }^{2}} { \left( \arctan \nolimits \frac{{w}^{gt}}{{h}^{gt}} -\arctan \nolimits \frac{w}{h} \right) }^{2}.\end{eqnarray*}

CIoU Loss primarily consolidates bounding box regression metrics such as centroid distance, intersection ratio, and aspect ratio. However, it does not explicitly address aligning the orientation between predicted and real bounding boxes. Additionally, when the width and height of bounding boxes *B* and *B*^*gt*^ converge to $ \left\{ \left( w=k{w}^{gt},h=k{h}^{gt} \right) \left\vert k\in {R}^{+} \right. \right\} $, the width and height of the bounding box regression *B* cannot simultaneously increase or decrease. This phenomenon leads to delayed convergence of the predicted bounding box during training, thereby hindering the real-time capabilities of the detection method.

**Figure 2:**
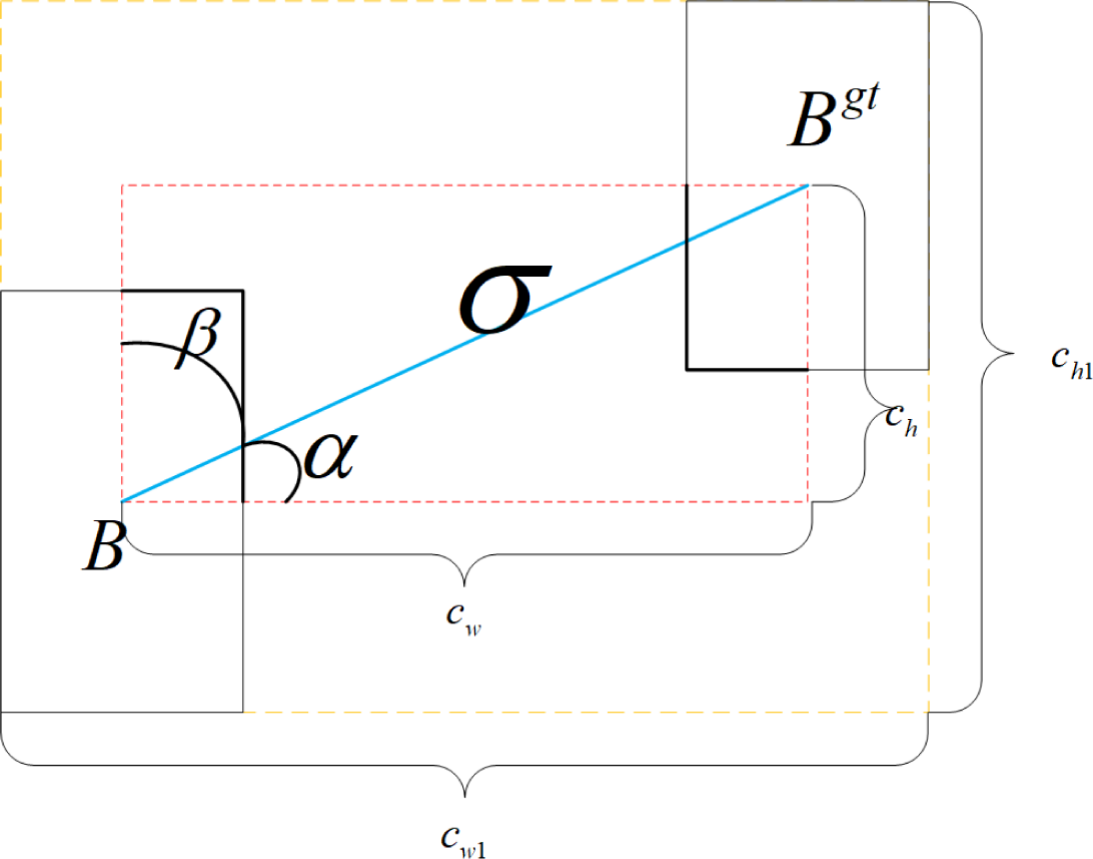
The concepts of SIoU Loss regression.

SIoU Loss is composed of Angle Loss Λ, Distance Loss, Shape Loss, and IoU Loss. The Angle Loss Λ is defined as follows:

(4) \begin{eqnarray*}\Lambda =1-2\text{*}{\sin \nolimits }^{2} \left( \arcsin \nolimits \left( \frac{{\text{c}}_{h}}{\sigma } \right) - \frac{\pi }{4} \right) =\cos \nolimits \left( 2\text{*} \left( \arcsin \nolimits \left( \frac{{\text{c}}_{h}}{\sigma } \right) - \frac{\pi }{4} \right) \right) ~\end{eqnarray*}

where $\sigma =\sqrt{{ \left( {b}_{{c}_{x}}^{gt}-{b}_{{c}_{x}} \right) }^{2}\text{+}{ \left( {b}_{{c}_{y}}^{gt}-{b}_{{c}_{y}} \right) }^{2}}$ and ${c}_{h}=\max \left( {b}_{{c}_{y}}^{gt},{b}_{{c}_{y}} \right) -\min \left( {b}_{{c}_{y}}^{gt},{b}_{{c}_{y}} \right) $; *σ* represents the distance from the center point of bounding boxes *B* and *B*^*gt*^; *c*_*h*_ denotes the disparity in height between the center point of bounding boxes *B* and *B*^*gt*^; *b*_*c*_*x*__, *b*_*c*_*y*__, ${b}_{{c}_{x}}^{gt}$, ${b}_{{c}_{y}}^{gt}$ are the center coordinates of bounding boxes *B* and *B*^*gt*^; $ \frac{{c}_{h}}{\sigma } $ is the opposite side of the right triangle relative to the hypotenuse $\sin \left( \alpha \right) $; In the training process, when $\alpha > \frac{\pi }{4} $, Λ takes *β*, otherwise, it takes *α*. The distance Loss can be expressed as:

(5) \begin{eqnarray*}\Delta =\sum _{\text{t=x},y} \left( 1-{\text{e}}^{-\gamma {\rho }_{t}} \right) =2-{\text{e}}^{-\gamma {\rho }_{x}}-{\text{e}}^{-\gamma {\rho }_{y}}~\end{eqnarray*}

where ${\rho }_{x}={ \left( \frac{{b}_{{c}_{x}}^{gt}-{b}_{{c}_{x}}}{{c}_{w1}} \right) }^{2}$, ${\rho }_{y}={ \left( \frac{{b}_{{c}_{y}}^{gt}-{b}_{{c}_{y}}}{{c}_{h1}} \right) }^{2}$, *γ* = 2 − Λ,and *c*_*w*1_, *c*_*h*1_ are the width and height of the smallest outer rectangle of bounding boxes *B* and *B*^*gt*^, respectively. The shape loss is defined in the form below:

(6) \begin{eqnarray*}\Omega ={\sum _{\text{t=w},h} \left( 1-{\text{e}}^{-{\text{w}}_{t}} \right) }^{\theta }={ \left( 1-{\text{e}}^{-{\text{w}}_{w}} \right) }^{\theta }+{ \left( 1-{\text{e}}^{-{\text{w}}_{h}} \right) }^{\theta }~\end{eqnarray*}

where ${\text{w}}_{\text{w}}= \frac{ \left\vert \text{w-}{\text{w}}^{gt} \right\vert }{\max \left( \text{w},{\text{w}}^{gt} \right) } $, ${\text{w}}_{h}= \frac{ \left\vert \text{h-}{\text{h}}^{gt} \right\vert }{\max \left( \text{h,}{\text{h}}^{gt} \right) } $; w, h, w^*gt*^, and h^*gt*^ correspond to the width and height of bounding boxes *B* and *B*^*gt*^, respectively. *θ* governs the degree of attention given to shape loss. To prevent an excessive focus on shape loss and minimize the displacement of the prediction bounding box, the value range of *θ* was set from 2 to 6. SIoU Loss is defined as follows, and its regression principle is shown in Fig. 2.

(7) \begin{eqnarray*}{L}_{SI\text{o}U}=1-I\text{o}U\text{+} \frac{\Delta \text{+}\Omega }{2} .\end{eqnarray*}

Given that the angle loss $\cos \left( 2\text{*} \left( \arcsin \left( \frac{{\text{c}}_{h}}{\sigma } \right) - \frac{\pi }{4} \right) \right) $ of $\arcsin \left( \frac{{\text{c}}_{h}}{\sigma } \right) - \frac{\pi }{4} =\alpha - \frac{\pi }{4} $ and $\beta = \frac{\pi }{2} -\alpha $ become identical following the cos function, the angle loss is minimized when *α* = 0 and maximized when it approaches *π*/4. This facilitates quicker movement of the prediction bounding boxes towards the nearest axis. Consequently, subsequent regression only necessitates *X* or *Y* coordinates, leading to a significant improvement in both model detection accuracy and training speed.

The loss function adopted in this article enables faster and more effective alignment of predicted bounding boxes with actual boxes compared to the loss function utilized in the algorithm outlined in Cai et al. (2021). This facilitates expedited training on large datasets, reduces time costs, and enhances the labeling accuracy of predicted bounding boxes on image instances. While the YOLOv6 algorithm employs an anchor-free SIoU Loss, this article utilizes an anchor-based SIoU Loss. Although the anchor-based approach marginally affects detection speed, it notably enhances detection accuracy. In summary, the loss function employed in this study is better suited for intelligent driving application scenarios.

### Optimization of the detection head

**Figure 3:**
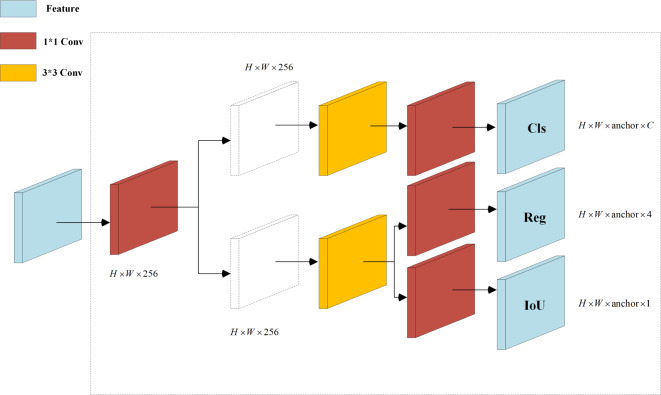
Decoupled heads diagram.

The original YOLOv5 detection algorithm adopts a coupled head format, as illustrated in Fig. 1, which outputs classification and regression tasks after a convolution layer. However, this approach leads to a conflict between classification, which emphasizes the resemblance of extracted features to specific categories, and regression, which focuses on adjusting predicted bounding box parameters based on differences from actual coordinates. To resolve this problem, Ge et al. (2021) introduced YOLOX with a decoupled head (DH) architecture. This architecture begins with a 1 × 1 convolution to alter the channel number to 256 before branching into two paths for classification and regression, with an additional IoU branch under the regression path. Each path undergoes two 3 × 3 convolution layers and a 1 × 1 convolution layer, resulting in separate heads for classification and regression. Studies Ge et al. (2021) demonstrated that DH enhances training speed and accuracy by resolving task conflict. However, the performed experiments indicate that while DH improves performance, it also increases the parameter count and floating-point operations (Gflops), resulting in higher memory usage and diminished real-time capability of the network. Therefore, this article introduces an enhanced decoupled head, named DHs, which replaces the network’s Head component, as illustrated in Fig. 3.

Figure 3 reveals that the input features *H* × *W* × *C* undergo an initial 1 × 1 convolution to compress the channel number to 256. Subsequently, in both parallel branches, one 3 × 3 convolution is removed to reduce the number of parameters. Finally, a 1 × 1 convolution is employed to extract features for classification and regression tasks. Compared to the coupled head structure of YOLOv5, the classification detection head in the DH improves the prediction of positive samples, while the regression detection head enhances the accuracy of bounding box regression. Ultimately, experimental results demonstrate that the developed DHs, despite reducing a significant number of parameters and floating-point operations, still maintain the advantages of the DH architecture.

In the algorithm outlined in Cai et al. (2021), the number of coupled detection heads was increased from three to five, resulting in a significant enhancement in the precision of predictions for small objects. In contrast, the proposed algorithm replaces three coupled detection heads with decoupled heads, thereby improving the prediction accuracy for small objects while concurrently reducing the number of parameters and the volume of floating-point operations. In comparison to the decoupled heads utilized in the YOLOX algorithm, the decoupled heads in the proposed algorithm maintain detection accuracy while substantially reducing the number of parameters and floating-point operations. Consequently, this accelerates the detection rate and renders it more suitable for practical application scenarios.

### Integration of lightweight global attention mechanism

**Figure 4:**
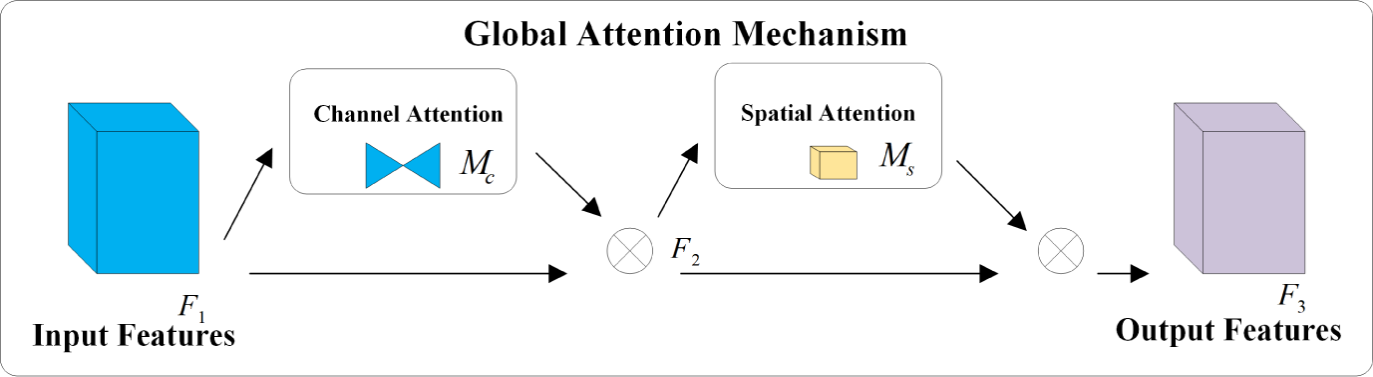
Structure of the GAM network.

The concept of the attention mechanism originated in the field of visual imagery and gained prominence with an article published by Google DeepMind (Mnih, Heess & Graves, 2014). By integrating the attention mechanism into the image processing workflow of computers, neural networks can emulate the human visual cognitive requirement of rapidly focusing on key information while neglecting less important data. This approach significantly conserves computing resources. Squeeze-and-Excitation Network (SeNet) focuses on the differences in channel information, adaptively adjusting the model based on the attention weights of each channel. However, this can lead to inefficiency issues when suppressing important information (Hu, Shen & Sun, 2018). The Convolutional Block Attention Module (CBAM) serially integrates attention weights in both channel and spatial dimensions on top of the input features, while the Bottleneck Attention Module (BAM) processes channel and spatial dimensions in parallel. However, both overlook the interaction between channel and spatial dimensions, losing cross-dimensional information (Woo et al., 2018; Park et al., 2020). Considering the importance of interactions between cross-dimensional information, the Triplet Attention Module (TAM) aims to enhance network efficiency by leveraging attention weights between channel, spatial width, and height dimensions. Yet, the attention operation is still applied to only two dimensions, not three (Misra et al., 2021). To amplify the interaction between the three dimensions, this article utilizes an attention mechanism called Global Attention Mechanism (GAM) capable of capturing important features in all three dimensions. Similar to CBAM, the overall structure employs a sequential arrangement of channel and spatial attention modules. The distinctions include not only the elimination of pooling operations to preserve as much information as possible but also the incorporation of an MLP to reduce the number of parameters. The specific network structure is depicted in Fig. 4 and mathematically expressed in Eqs. (8) and (9), where *F*_1_ represents the input feature, *F*_2_ denotes the intermediate state, and *F*_3_ is the output feature, while *M*_*c*_ and *M*_*s*_ respectively represent the channel and spatial attention, and ⊗ denotes the element-wise multiplication.

(8) \begin{eqnarray*}{F}_{2}={M}_{c} \left( {F}_{1} \right) \otimes {F}_{1}~\end{eqnarray*}

(9) \begin{eqnarray*}{F}_{3}={M}_{s} \left( {F}_{2} \right) \otimes {F}_{2}.\end{eqnarray*}

In Fig. 4, the input feature *F*_1_ will initially pass through two branches. The feature of the upper branch will undergo the channel attention module and then multiply with the input feature *F*_1_ of the bottom branch to obtain *F*_2_. Subsequently, *F*_2_ will go through two branches again. The feature of the upper branch will be multiplied with the input feature *F*_2_ of the bottom branch through the spatial attention module to obtain the output feature *F*_3_.

**Figure 5:**
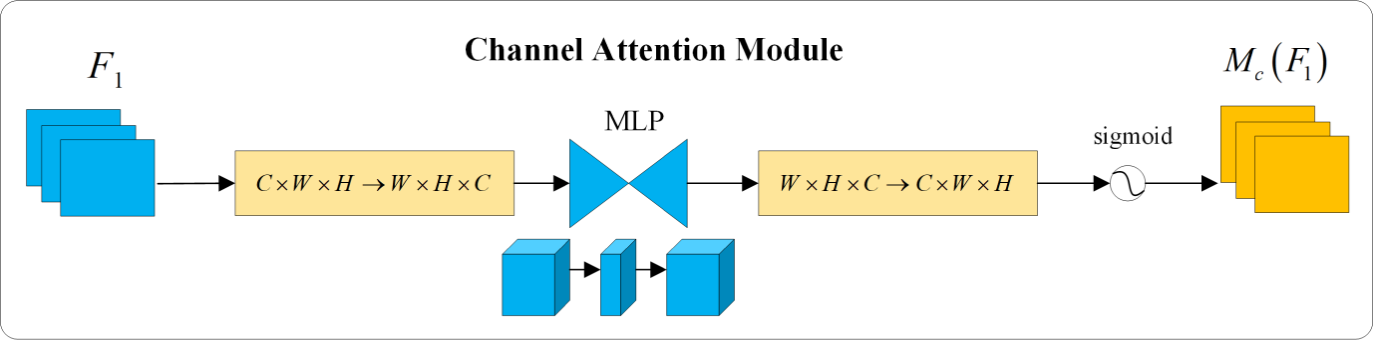
Channel attention module.

The channel attention module removes the max pooling operation to preserve the integrity of information. It employs a permutation approach to retain information across three dimensions. Subsequently, it utilizes a two-layer multi-layer perceptron (MLP) with an encode-decode structure, having a reduction ratio of *γ*, to amplify the dependency relationship between channels and spatial dimensions. This process is specifically illustrated in Fig. 5.

**Figure 6:**
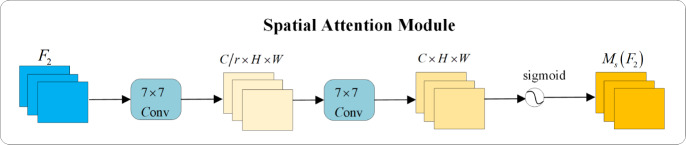
Spatial attention module.

In the spatial attention module, to focus on spatial information, two convolutional layers are utilized for the extraction of spatial information. A channel reduction ratio of *γ* is employed to decrease the number of parameters. This process is schematically illustrated in Fig. 6.

**Figure 7:**
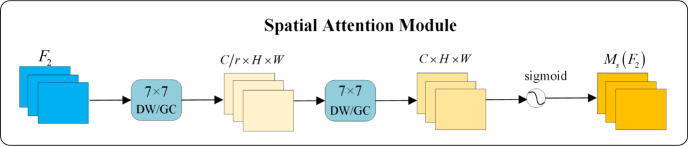
Improved attention mechanism structure diagram.

When employing the GAM, a notable drawback arises from the significant increase in the number of parameters due to the two convolutional layers in its spatial attention module. To resolve this issue, the standard convolutions are replaced with group convolutions (GC) and depthwise separable convolutions (DW). The process is schematically illustrated in Fig. 7.

• Group Convolution divides the input feature map and convolution kernels into separate groups, executing convolution operations within these groups to diminish the number of parameters. Suppose the input feature map size is *C* × *H* × *W* and it undergoes *N* standard *K* × *K* convolutions. In that case, the total number of parameters is *N* × *C* × *K* × *K*. With Group Convolution, the standard convolution is segmented into *g* groups, with each convolution size being $ \frac{C}{g} \times K\times K$, resulting in the total number of parameters being $N\times \frac{C}{g} \times K\times K$. Consequently, the final parameter count is $ \frac{1}{g} $ of the original. In this article, the group convolution is divided into four groups, and the modified attention mechanism with group convolution is called GAMGC.
• Depthwise Separable Convolution breaks down a standard convolution into a depthwise convolution and a 1 × 1 pointwise convolution. Unlike standard convolutions that consider semantic information across all channels, each kernel in a depthwise convolution concentrates on a single channel. The size of the depthwise convolution is *K* × *K* × 1, resulting in a parameter count of *K* × *K* × 1 × *N*. The pointwise convolution, utilizing a 1 × 1 × *C* kernel, extracts semantic information between channels, with a parameter count of 1 × 1 × *C* × *N*. Thus, the total number of parameters for Depthwise Separable Convolution is *K* × *K* × 1 × *N* + 1 × 1 × *C* × *N*, which is even smaller than that of Group Convolution. In this article, the attention mechanism replaced by Depthwise Separable Convolution is termed GAMDW.

In Fig. 7, the two convolutions within the spatial attention module are substituted with DWs and GCs to decrease parameter counts. Experimental analysis demonstrates that these modifications not only reduce the number of parameters but also improve the extraction of essential semantic information. It is worth noting that the performance of GAMGC surpasses that of GAMDW, leading to the selection of GAMGC for incorporation into the YOLOv5 network in this research.

In the algorithm outlined in Zhu et al. (2021), the CBAM attention mechanism is integrated into the neck network of YOLOv5, effectively diminishing the influence of ambiguous information in images on the algorithm’s detection performance. This integration enables the algorithm to concentrate on vital semantic information across two dimensions. Conversely, in this article, the GAMGC is incorporated into both the backbone and neck networks of YOLOv5. This methodology facilitates the capture of critical semantic information across three dimensions, thereby improving target detection accuracy while keeping the parameter count and floating-point operations low.

## Experiments

### Experimental dataset & evaluation index & experimental environment

**Figure 8:**
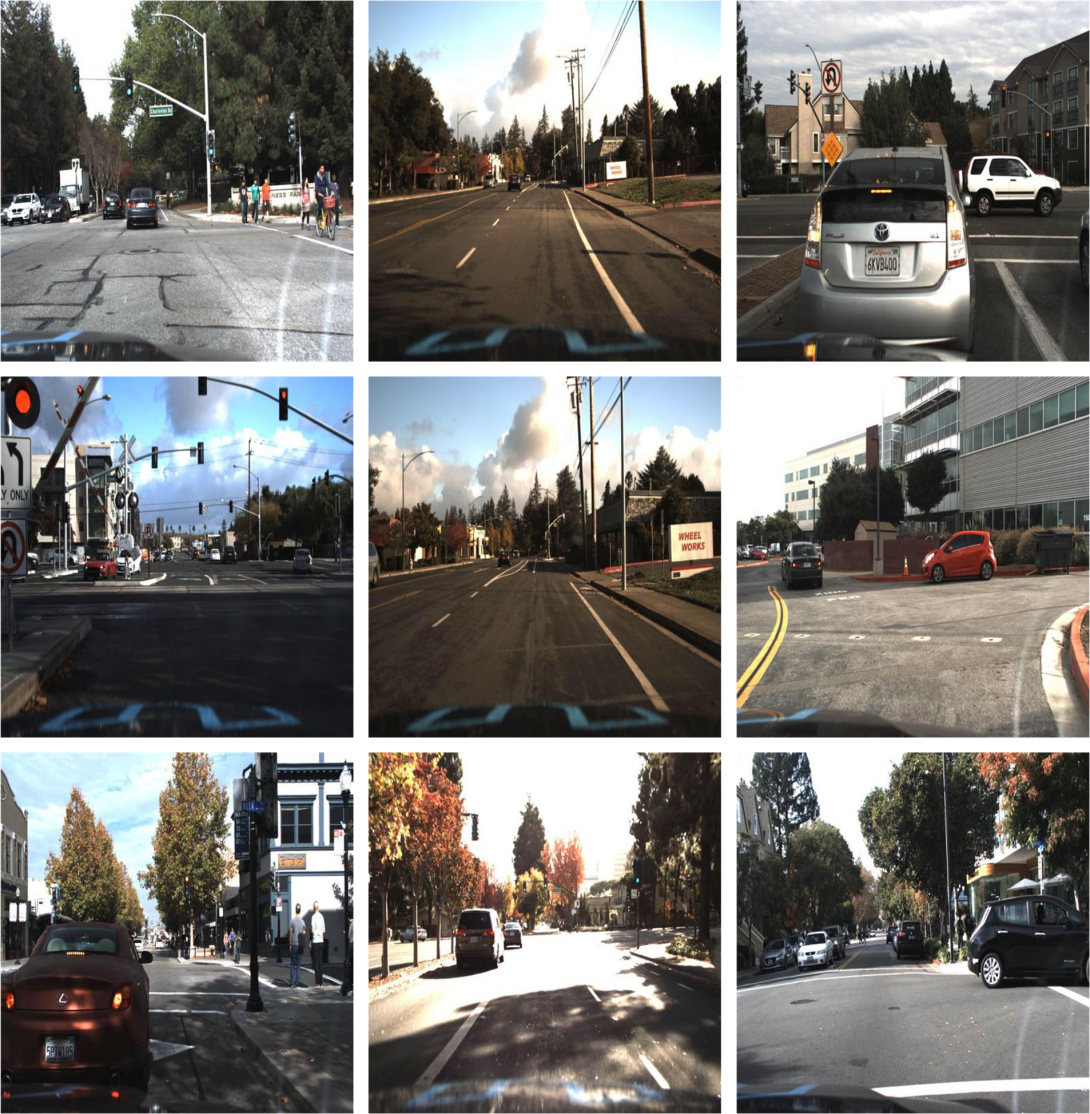
Sample images extracted from datasets.

The datasets chosen for this experiment include the Udacity Self Driving Car, BDD100K, and KITTI datasets. The Udacity dataset provides 2D annotations for continuous video images, covering 11 categories: biker, car, pedestrian, traffic light, traffic light-green, traffic light-green left, traffic light-red, traffic light-red left, traffic light-yellow, traffic light-yellow left, and truck. However, to maintain consistency and reduce variability in model results, the category traffic light-yellow left, which has a limited number of labels, was excluded, resulting in 10 categories for the experiment. This dataset comprises a total of 29,800 images with a resolution of 512 × 512, split into training and testing sets in a 9:1 ratio, with 26,579 images for training and 3,221 for testing. The BDD100K dataset consists of 70 k images for training and 10 k for validation. Due to the inadequate number of labels in the train category, this dataset was divided into 12 categories: Person, Rider, Car, Bus, Truck, Bike, Motor, Traffic Light_Green (TL_G), Traffic Light-Red (TL_R), Traffic Light-Yellow (TL_Y), Traffic Light-None (TL_N), and Traffic Sign (TS). The KITTI dataset comprises 7,481 images for training and 7,518 for validation. Since the validation set lacks labels, the 7,481 training images were divided into training and validation sets in an 8:2 ratio. The dataset was categorized into three classes: Car, Pedestrian, and Cyclist. Some images extracted from these databases are depicted in Fig. 8.

The evaluation metrics employed in the experiments include precision (P), recall (R), accuracy (mAP@. 5), number of parameters (Parameters), floating-point operations (GFLOPS), and frame rate (FPS). These metrics are defined as follows:

(10) \begin{eqnarray*}P= \frac{TP}{TP+FP} ~\end{eqnarray*}

(11) \begin{eqnarray*}R= \frac{TP}{TP+FN} ~\end{eqnarray*}

(12) \begin{eqnarray*}mAP@.5= \frac{\sum _{i=1}^{{N}_{\text{class}}}\int \nolimits \nolimits _{0}^{1}{P}_{i}{R}_{i}dR}{{N}_{class}} ~\end{eqnarray*}

where *TP* refers to the number of true positive detections with an IoU greater than a specified threshold, *FP* refers to the number of false positive detections with an IoU less than or equal to this threshold, and *FN* indicates the number of undetected true boxes. Furthermore, @.5 denotes an IoU threshold of 0.5, *N*_*class*_ represents the total number of classes, $\int \nolimits _{0}^{1}{P}_{i}{R}_{i}dR$ represents the accuracy of the ith class target, and *mAP* stands for the mean average precision across all class targets.

The experimental setup and server environment are as follows: the GPU model utilized is an NVIDIA RTX 3090 with 24 GB of VRAM, while the CPU model is an Intel Xeon Platinum 8,350 C. The software stack includes PyTorch version 1.10.0, Python 3.8, and CUDA 11.3, operating on the Ubuntu 20.04 as operating system. The training parameters are configured as follows: the input image size is set to 640 × 640, with a maximum of 300 iterations and a batch size of 32. The optimizer employed is SGD with a momentum of 0.937 and a weight decay coefficient of 0.0005. The initial learning rate is established at 0.01, with dynamic adjustments according to the cosine annealing algorithm, leading to a final learning rate of 0.002.

### Analysis of experimental results

In the research conducted, an ablation experiment methodology was employed on the Udacity Self Driving Car dataset, mirroring the approach used by YOLOX. This methodology involved a strategy of parallel insertion of each module, aiming to assess the individual contribution of each module to the overall performance of the model. By utilizing this parallel ablation approach, the study aimed to precisely determine the effectiveness of each module and its impact on fine-tuning the algorithm. This methodology ensures that the effects of each module are evaluated independently, providing a direct means to observe how each component influences the system’s accuracy and efficiency in real-world intelligent driving scenarios. The experimental results are presented in Table 1.

**Table 1:** Ablation experiment on Udacity self driving car dataset.

Methods	P	R	mAP@.5 (%)	Parameters (MB)	GFLOPS	FPS
*Baseline*	91.2	74.5	86.1	6.76	16.6	55.6
+*SIoULoss*	90.2	75.6	86.8	6.76	16.6	62.5
+*DH*	80.1	87.1	87.4	13.73	57.1	34.5
+*DHs*	73.1	88.2	86.8	10.35	37.2	41.7
+*GAM*	94	76.2	87.9	28.65	74.3	21.3
+*GAMGC*	89.8	76.2	87.1	12.57	31.9	41.7
+*GAMDW*	92.3	75.5	87.2	7.76	19.3	47.6
SIoU+DHs+GAMGC	76	88.6	88.3	16.16	52.6	30.3

Table 1 indicates that the YOLOv5 algorithm achieves a detection accuracy of 86.1% for road scenes, but with a relatively low recall rate. Conversely, combining SIoU Loss, DHs, and GAMGC algorithms enhances detection accuracy while sustaining the detection rate. Specifically, for road scene detection, the accuracy reached 88.3%, marking a 2.2% increase in mAP@.5.

These enhancements were sequentially integrated into the YOLOv5 network, leading to notable improvements in detection accuracy.

• After integrating the SIoU Loss, there was a notable improvement in various metrics. Specifically, the recall rate, mAP@.5, and the frame rate increased by 1.1%, 0.7%, and 6.9 FPS, respectively. This indicates that the angle loss component within the SIoU Loss plays a crucial role in refining the regression of predicted bounding boxes, particularly benefiting categories with fewer labels. The effectiveness of SIoU Loss may be attributed to its comprehensive design, which accounts for not only the size, position, and aspect ratio of bounding boxes but also their orientation. By considering these geometric properties, SIoU Loss enables a more accurate alignment of predicted boxes with actual ones, especially in scenarios involving closely packed or partially obscured objects. Through better management of angular discrepancies between predicted and actual boxes, SIoU Loss effectively enhances overall precision and detection speed.
• Following the integration of the DH, there was a notable enhancement in various metrics. Specifically, mAP@.5 and the recall rate increased by 1.3% and 12.6%, respectively, indicating a promising accuracy in predicting positive samples and precision in bounding box regression. However, this improvement came at the cost of increased model parameters and computational load, thereby elevating the model’s complexity. To address this challenge, a lightweight version of the decoupled head, termed DHs, was developed. Experimental validation revealed that after incorporating DHs, although there was a slight reduction in mAP@.5 (a decrease of 0.6% compared to +DH, but an improvement of 0.7% compared to the original network), there was a substantial reduction of 24.6% in the model’s parameter count and a decrease of 19.9 GFLOPS in computational load (floating-point operations). Additionally, there was an increase in frame rate by 7.2 FPS. These results demonstrate the capability of DHs to significantly reduce computational costs and enhance processing speed without compromising detection performance significantly, thereby validating the effectiveness and practicality of the optimization strategy employed.
• According to Table 1, the investigation highlights the effectiveness of GAM, GAMGC, and GAMDW methods. The results demonstrate that each method notably enhances the accuracy of the model. GAM utilizes large 7 × 7 convolutions to enhance the model’s ability to capture intricate features. While achieving a 1.8% improvement in mAP@.5 compared to the original network, it also substantially increases the model’s parameters and floating-point operations, thereby impacting the frame rate and detection efficiency. To resolve this challenge, GAMGC employs group convolution, processing input feature maps in groups to reduce the model’s parameters and computational load while retaining sufficient feature learning capabilities. This enhancement enables GAMGC to improve mAP@.5 over the original network while significantly reducing parameters by 56% and floating-point operations by 42.4 GFLOPS, with a substantial frame rate increase of 20.4 FPS. This observation demonstrates GAMGC’s effectiveness in reducing the model’s burden while maintaining detection accuracy. Similarly, GAMDW, by utilizing depthwise separable convolutions, decomposes traditional convolutions into depthwise and pointwise convolutions, further decreasing parameter count and computational complexity. GAMDW achieves a 1.1% mAP@.5 improvement over the original network and outperforms GAM in reducing model parameters by 72.9% and floating-point operations by 55 GFLOPS, with a frame rate increase of 26.3 FPS. The adoption of depthwise separable convolutions not only significantly reduces computational load but also enhances detection accuracy.

While the combination of GAMDW+SIoU Loss+DHs reached an mAP@.5 performance of 87.5%, slightly lower than the combination with GAMGC, this discrepancy may be attributed to GAMDW’s reduction in model parameters and computational complexity through depthwise separable convolutions. Although this design improves computational efficiency, it may not capture complex features as effectively as GAMGC. Consequently, when combined with optimization techniques aimed at improving detection accuracy, GAMDW’s limitations might hinder these techniques from reaching their full potential, resulting in a less pronounced overall performance improvement compared to GAMGC. Given that the performance of GAMDW combined with SIoU Loss and DHs, although superior to the original network, still falls short compared to GAMGC, the choice was made to integrate GAMGC into the YOLOv5 network. This decision allows for more effective utilization of the performance enhancements brought by SIoU Loss and DHs while reducing the computational burden. As a result, high accuracy is achieved while optimizing the model’s computational efficiency and detection speed.

**Figure 9:**
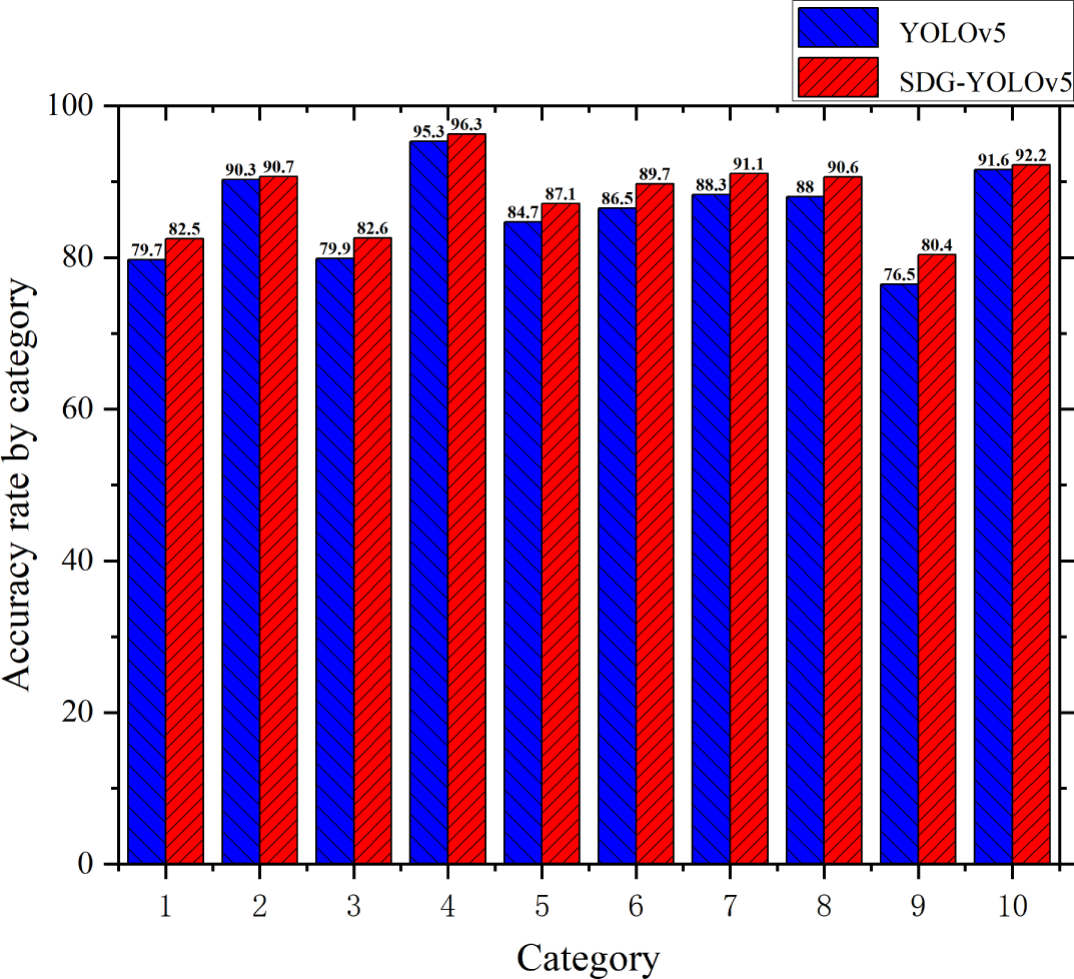
Accuracy chart before and after training on Udacity Self Driving Car dataset.

To validate the advantages of the proposed SDG-YOLOv5 over the original YOLOv5 model in terms of detection accuracy for each category on the Udacity Self Driving Car dataset, a category-specific comparative experiment was conducted. This approach aimed to reflect the performance improvements of SDG-YOLOv5 across different categories, allowing for an accurate assessment of the enhanced model’s performance and adaptability in complex scenarios. Through this category-based comparison, the changes in detection accuracy were explored for each category, demonstrating the overall performance enhancement of the model and revealing its strengths and potential areas for improvement in specific categories. The training results of mAP@.5 before and after the improvements are depicted in Fig. 9. In this figure, the average precision (AP) for the categories biker, car, pedestrian, traffic light, traffic light-green, traffic light-green left, traffic light-red, traffic light-red left, traffic light-yellow, and truck are represented by the numbers 1–10, respectively.

Figure 9 reveals notable improvements in detection accuracy across various categories in the enhanced network. Specifically, the ‘biker’, ‘car’, and ‘pedestrian’ categories increased by 2.8%, 0.4%, and 2.7%, respectively. Among the categories related to traffic signals, ‘trafficLight’, ‘trafficLight-Green’, and ‘trafficLight-GreenLeft’ increased by 1%, 2.4%, and 3.2% , respectively, while ‘trafficLight-Red’, ‘trafficLight-RedLeft’, and ‘trafficLight-Yellow’ improved by 2.8%, 2.6%, and 3.9%, respectively. Moreover, it is observed that the detection accuracy of ‘truck’ category is enhanced by 0.6%. These improvements may be attributed to the synergistic effects of several optimization techniques. SIoU Loss, by considering the orientation of bounding boxes, enhanced geometric alignment, leading to improved detection accuracy. The lightweight DHs effectively reduced model complexity while maintaining sufficient feature representation capabilities. Additionally, the attention mechanism (GAMGC) played a crucial role in recognizing small targets like traffic lights by focusing on key features. By incorporating these optimization measures, the SDG-YOLO algorithm achieved performance enhancements across all categories of the Udacity Self Driving Car dataset, significantly improving the accuracy of multi-category target detection.

To evaluate the effectiveness and generalization capability of the proposed SDG-YOLOv5 algorithm, this study conducts comparative experiments on both YOLOv5 and SDG-YOLOv5 algorithms using the BDD100K and KITTI datasets. This experimental approach was chosen because these datasets represent distinct driving scenes and challenges, enabling a comprehensive evaluation of SDG-YOLOv5’s detection accuracy across diverse environments and categories. Through such comparative experiments, the performance of SDG-YOLOv5 can be compared with the original YOLOv5 model in different scenarios. Consequently, the algorithm’s generalization ability can be evaluated, thereby ensuring its reliability and effectiveness in practical applications. Table 2 presents the detection accuracies of SDG-YOLOv5 and YOLOv5 over BDD100K and KITTI datasets.

**Table 2:** Accuracy comparison between YOLOv5 and SDG-YOLOv5 on datasets BDD100K and KITTI.

**BDD100K**	Person	Rider	Car	Bus	Truck	Bike	Motor	TL_G	TL_R	TL_Y	TL_N	TS	mAP@.5(%)
YOLOv5	57.1	41.5	75.5	57.8	59.1	44.6	40.5	59.8	52.7	21.7	48.4	64	51.9
Ours	60.4	44.5	77.7	60	60.6	48.2	43.6	64.3	56.7	24.6	54.7	67.7	55.3
**KITTI**	**Car**	**Pedestrian**	**Cyclist**										**mAP@.5(%)**
YOLOv5	98.1	88	94.5										93.6
Ours	98.4	89.6	96										94.6

Table 2 indicates that the mAP@.5 achieved by the YOLOv5 algorithm was 51.9% and 93.6%, respectively. In contrast, the mAP@.5 for the SDG-YOLOv5 algorithm reached 55.3% and 94.6%, representing an improvement in detection accuracy of 3.4% and 1.0% over the original algorithm, respectively. A detailed analysis of the AP values for each category detected by YOLOv5 and SDG-YOLOv5 demonstrates that SDG-YOLOv5 not only achieved significant accuracy enhancements in conventional large target categories such as ‘Car’, ‘Bus’, ‘Pedestrian’, and ‘Cyclist’, but also realized substantial accuracy improvements in small target categories, including different states of traffic lights (‘TL_G’, ‘TL_R’, ‘TL_Y’, ‘TL_N’). These findings effectively validate the efficiency and superiority of the SDG-YOLOv5 algorithm in processing complex traffic scenes, as well as its exceptional generalization capability across datasets.

To demonstrate the superiority of the algorithm proposed in this article, a comparison of its detection accuracy was conducted with current mainstream object detection algorithms on the Udacity Self Driving Car dataset. The experimental results are presented in Table 3. By selecting widely recognized and representative algorithms in this field as benchmarks, the aim is to comprehensively explore the performance advantages of the algorithm in processing complex road scenes. Moreover, this comparison allows for a more objective evaluation of its position and value in the current object detection technology landscape.

**Table 3:** Performance comparison on the dataset Udacity Self Driving Car.

*Detector*	Backbone	mAP@.5 (%)	FPS
*FasterR* − *CNN* (Ren et al., 2017)	ResNet-50	78.6	10.7
*AD* − *FasterR* − *CNN* (Zhou et al., 2021b)	AD-ResNet-50	82.3	6
*SSD* (Liu et al., 2016)	VGG-16	52.1	21.8
*YOLOv*3 (Redmon & Farhadi, 2018)	DarkNet-19	81.3	49.3
*YOLOv*4 − *CSPResNeXt* (Priya, Rajalakshmi & Jebamalar, 2022)	CSPResNeXt	83.4	52.3
*YOLOv*5	C3	86.1	55.6
*TPH* − *YOLOv*5 (Zhu et al., 2021)	C3-Transformer	87.9	36.3
*BIGA* − *YOLO* (Liu et al., 2023a)	C3-Ghost	84.1	114.9
*CF* − *YOLOX* (Wu, Yan & Wang, 2023)	C3-CBAM-G	85.8	75.1
*YOLOv*8	C2F	87.3	87.1
SDG-YOLOv5 (Ours)	C3	88.3	30.3

Table 3 presents a comparative analysis between SDG-YOLOv5 and existing mainstream algorithms on the Udacity Self Driving Car dataset. In contrast to traditional two-stage algorithms like Faster R-CNN and its enhanced version AD-Faster R-CNN, SDG-YOLOv5 demonstrates an improvement in the mAP@.5 metric by 9.7% and 6%, respectively, along with a boost in frame rate by 19.6 FPS and 24.3 FPS, respectively. This advancement not only demonstrates SDG-YOLOv5’s superiority over traditional two-stage algorithms in effectively integrating spatial and contextual information but also reflects its progress in frame rate, a crucial factor for real-time applications such as intelligent driving. When compared to the single-stage algorithm SSD, SDG-YOLOv5 achieves a notable enhancement of 36.2% in the mAP@.5 metric and an 8.5 FPS increase in frame rate. Additionally, compared to YOLOv3 and YOLOv4-CSPResNeXt, SDG-YOLOv5 respectively exhibits a 7% and 4.9% increase in mAP@.5, demonstrating its effectiveness in improving detection accuracy. In comparison to CF-YOLOX, which incorporates the CBAM attention mechanism, SDG-YOLOv5 has demonstrated a 2.5% enhancement in the mAP@.5 metric, reflecting its effectiveness in improving context-aware features. Additionally, when compared to TPH-YOLOv5, which integrates a self-attention mechanism, SDG-YOLOv5 exhibited a 0.4% increase in the mAP@.5 evaluation metric. While self-attention mechanisms are recognized for improving a model’s feature learning capabilities, they often require longer training periods. The enhancements integrated into SDG-YOLOv5 enable it to notably boost detection accuracy while reducing the training duration, which is particularly vital for applications like intelligent driving requiring rapid iteration and deployment. Against BIGA-YOLO, utilizing lightweight Ghost modules, SDG-YOLOv5 has displayed a 4.2% rise in the mAP@.5 metric. Furthermore, in comparison to the latest YOLOv8, SDG-YOLOv5 showcased a 1% improvement in the mAP@.5 metric, affirming its superior detection accuracy in intelligent driving scenarios. The analysis of the data presented in the table indicates that the proposed algorithm, SDG-YOLOv5, has achieved the highest accuracy while maintaining a frame rate of 30.3 FPS, thus meeting the requirements for real-time detection. Although the detection speed of SDG-YOLOv5 may be slightly slower compared to some mainstream algorithms, prioritizing increased detection accuracy while ensuring real-time performance is deemed more critical in the context of intelligent driving applications.

**Figure 10:**
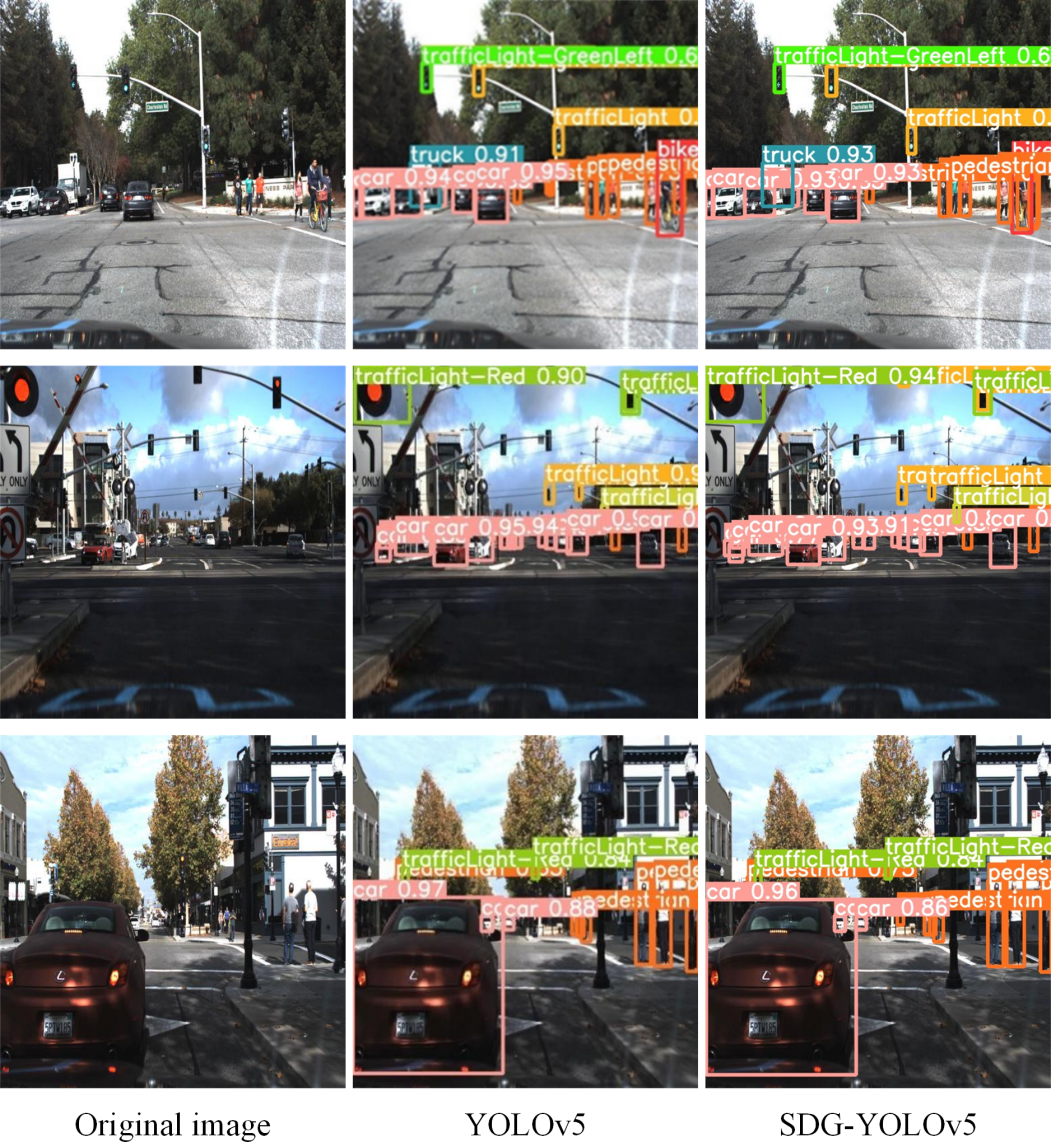
Comparison of test results obtained from different algorithms.

To evaluate the efficiency of the proposed algorithm, random tests were conducted on the Udacity Self Driving Car dataset using the model loaded with training-exported weights. The test results depicted in Fig. 10 reveal that the original network encounters challenges in accurately detecting certain object categories. Specifically, it demonstrates tendencies to misclassify crucial targets like traffic lights and pedestrians. Moreover, instances of missed detections are observed, where it fails to identify some small and distant objects, thus constraining its utility in traffic scenarios. In contrast, the SDG-YOLOv5 algorithm outlined in this article effectively tackles these issues. It adeptly identifies and precisely localizes targets across diverse road conditions, markedly enhancing the precision of object detection.

## Conclusion

The present study develops the SDG-YOLOv5 algorithm to achieve high real-time performance and precision in smart driving object detection scenarios. To this end, the CIoU Loss is replaced with SIoU Loss to accelerate network convergence, facilitating quicker and more accurate alignment of predicted bounding boxes with actual ones. Subsequently, lightweight DHs are introduced to replace the original detection head, effectively segregating classification and regression tasks and significantly reducing the number of parameters while maintaining accuracy compared to the traditional decoupled head. Furthermore, the lightweight GAMGC is incorporated into the original network, resulting in a notable reduction in the parameter count and floating-point operations compared to conventional attention mechanisms. The performed analyses demonstrate that the algorithm achieves mAP@.5 scores of 88.3%, 55.3%, and 94.6% on the Udacity Self Driving Car, BDD100K, and KITTI datasets, respectively, under testing conditions with an NVIDIA RTX 3090. Additionally, the detection speed is 30.3FPS, significantly enhancing the detection of road scenes while balancing real-time performance.

## Additional Information and Declarations

### Data Availability

The following information was supplied regarding data availability: The Udacity dataset is available at Zenodo: Lv, Z. (2024). udacity [Data set]. Zenodo. https://doi.org/10.5281/zenodo.10531694. The BDD100K dataset is available at Zenodo: Lv, Z. (2024). BDD100K [Data set]. Zenodo. https://doi.org/10.5281/zenodo.10820332. The KITTI dataset is available at Zenodo: Lv, Z. (2024). KITTI [Data set]. Zenodo. https://doi.org/10.5281/zenodo.10820379. The code is available at GitHub: https://github.com/yyyert/SDG-YOLOv5

## References

[ref-1] Bochkovskiy A, Wang C-Y, Liao H-Y (2020). YOLOv4: optimal speed and accuracy of object detection. ArXiv.

[ref-2] Cai Y, Luan T, Gao H, Wang H, Chen L, Li Y, Sotelo M, Li Z (2021). YOLOv4-5D: an effective and efficient object detector for autonomous driving. IEEE Transactions on Instrumentation and Measurement.

[ref-3] Du Y, Qin B, Zhao C, Zhu Y, Cao J, Ji Y (2022). A novel spatio-temporal synchronization method of roadside asynchronous MMW radar-camera for sensor fusion. IEEE Transactions on Intelligent Transportation Systems.

[ref-4] Ge Z, Liu S, Wang F, Li Z, Sun J (2021). YOLOX: exceeding YOLO series in 2021. ArXiv.

[ref-5] Girshick R, Donahue J, Darrell T, Malik J (2014). Rich feature hierarchies for accurate object detection and semantic segmentation.

[ref-6] He K, Zhang X, Ren S, Sun J (2015). Spatial pyramid pooling in deep convolutional networks for visual recognition. IEEE Transactions on Pattern Analysis and Machine Intelligence.

[ref-7] Hu J, Shen L, Sun G (2018). Squeeze-and-excitation networks.

[ref-8] Li C, Li L, Jiang H, Weng K, Geng Y, Li L, Ke Z, Li Q, Cheng M, Nie W, Li Y, Zhang B, Liang Y, Zhou L, Xu X, Chu X, Wei X, Wei X (2022). YOLOv6: a single-stage object detection framework for industrial applications. ArXiv.

[ref-9] Lin Q, Li S, Wang R, Wang Y, Zhou F, Chen Z, Guo N (2022). Research on small target detection technology based on the MPH-SSD algorithm. Computational Intelligence and Neuroscience.

[ref-10] Liu W, Anguelov D, Erhan D, Szegedy C, Reed S, Fu C-Y, Berg A, Leibe B, Matas J, Sebe N, Welling M (2016). SSD: single shot multibox detector. Computer Vision –ECCV 2016.

[ref-11] Liu J, Cai Q, Zou F, Zhu Y, Liao L, Guo F (2023a). BiGA-YOLO: a lightweight object detection network based on YOLOv5 for autonomous driving. Electronics.

[ref-12] Liu Y, Jiang D, Xu C, Sun Y, Jiang G, Tao B, Tong X, Xu M, Li G, Yun J (2023b). Deep learning based 3D target detection for indoor scenes. Applied Intelligence.

[ref-13] Liu X, Wu R, Wang R, Zhou F, Chen Z, Guo N (2022). Bearing fault diagnosis based on particle swarm optimization fusion convolutional neural network. Frontiers in Neurorobotics.

[ref-14] Luo G, Yuan Q, Li J, Wang S, Yang F (2022). Artificial intelligence powered mobile networks: from cognition to decision. IEEE Network.

[ref-15] Misra D, Nalamada T, Arasanipalai A, Hou Q (2021). Rotate to attend: convolutional triplet attention module.

[ref-16] Mnih V, Heess N, Graves A, Kavukcuoglu K (2014). Recurrent models of visual attention. Advances in neural information processing systems, volume 27.

[ref-17] Park J, Woo S, Lee J, Kweon I (2020). A simple and light-weight attention module for convolutional neural networks. International Journal of Computer Vision.

[ref-18] Priya S, Rajalakshmi J, Jebamalar G (2022). A novel method for object detection in autonomous driving system using cspresnext and YOLO-V4. International Journal of Early Childhood Special Education.

[ref-19] Redmon J, Divvala S, Girshick R, Farhadi A (2016). You only look once: unified, real-time object detection.

[ref-20] Redmon J, Farhadi A (2017). YOLO9000: better, faster, stronger.

[ref-21] Redmon J, Farhadi A (2018). YOLOv3: an incremental improvement. ArXiV.

[ref-22] Ren S, He K, Girshick R, Sun J (2017). Faster R-CNN: towards real-time object detection with region proposal networks. IEEE Transactions on Pattern Analysis and Machine Intelligence.

[ref-23] Wang C, Bochkovskiy A, Liao H (2023). YOLOv7: trainable bag-of-freebies sets new state-of-the-art for real-time object detectors.

[ref-24] Wang H, Xu Y, Wang Z, Cai Y, Chen L, Li Y (2023). CenterNet-Auto: a multi-object visual detection algorithm for autonomous driving scenes based on improved CenterNet. IEEE Transactions on Emerging Topics in Computational Intelligence.

[ref-25] Woo S, Park J, Lee J, Kweon I (2018). CBAM: convolutional block attention module.

[ref-26] Wu S, Yan Y, Wang W (2023). CF-YOLOX: an autonomous driving detection model for multi-scale object detection. Sensors.

[ref-27] Xu J, Park S, Zhang X, Hu J (2022a). The improvement of road driving safety guided by visual inattentional blindness. IEEE Transactions on Intelligent Transportation Systems.

[ref-28] Xu J, Zhang X, Park S, Guo K (2022b). The alleviation of perceptual blindness during driving in urban areas guided by saccades recommendation. IEEE Transactions on Intelligent Transportation Systems.

[ref-29] Zhang Y, Huang Y, Zhang Z, Postolache O, Mi C (2023). A vision-based container position measuring system for ARMG. Measurement and Control.

[ref-30] Zheng Z, Wang P, Liu W, Li J, Ye R, Ren D (2020). Distance-IoU loss: faster and better learning for bounding box regression. Proceedings of the AAAI Conference on Artificial Intelligence.

[ref-31] Zhou W, Liu J, Lei J, Yu L, Hwang J-N (2021a). GMNet: graded-feature multilabel-learning network for RGB-thermal urban scene semantic segmentation. IEEE Transactions on Image Processing.

[ref-32] Zhou Y, Wen S, Wang D, Mu J, Richard I (2021b). Object detection in autonomous driving scenarios based on an improved faster-RCNN. Applied Sciences.

[ref-33] Zhou W, Yu L, Zhou Y, Qiu W, Wu M-W, Luo T (2018). Local and global feature learning for blind quality evaluation of screen content and natural scene images. IEEE Transactions on Image Processing.

[ref-34] Zhu X, Lyu S, Xu W, Zhao Q (2021). TPH-YOLOv5: improved YOLOv5 based on transformer prediction head for object detection on drone-captured scenarios.

